# Switching to twice-yearly depemokimab from mepolizumab/benralizumab in severe asthma: a multicenter, randomized, double-blind, phase 3A clinical trial (NIMBLE)

**DOI:** 10.1093/ajrccm/aamag031

**Published:** 2026-02-11

**Authors:** Geoffrey Chupp, Hiroyuki Nagase, Dirk Skowasch, Gilles Devouassoux, Andréanne Côté, Daniel J Jackson, David J Jackson, Michael E Wechsler, Varsha Imber, John E McGinniss, Sherine O K, Peter Howarth, Ian D Pavord, the NIMBLE Study Investigators, Geoffrey Chupp, Hiroyuki Nagase, Dirk Skowasch, Gilles Devouassoux, Andréanne Côté, Daniel J Jackson, David J Jackson, Michael E Wechsler, Varsha Imber, John E McGinniss, Sherine O K, Peter Howarth, Ian D Pavord

**Affiliations:** Department of Internal Medicine, Yale School of Medicine, New Haven, CT, United States; Department of Medicine, Teikyo University School of Medicine, Tokyo, Japan; Department of Internal Medicine II, Cardiology, Pneumology, Angiology, University Hospital Bonn, Bonn, Germany; Service de Pneumologie, Hôpital de la Croix Rousse—HCL, CRISALIS F-CRIN INSERM Network, VIRPATH, Université Claude Bernard, Lyon, France; Department of Medicine, Institut Universitaire de Cardiologie et de Pneumologie de Quebec-Université, Laval, QC, Canada; Departments of Pediatrics and Medicine, University of Wisconsin–Madison, Madison, WI, United States; Guy’s Severe Asthma Centre, School of Immunology and Microbial Sciences, Guy’s Hospital, King’s College London, London, United Kingdom; Department of Medicine, National Jewish Health, Denver, CO, United States; Research & Development, GSK, London, United Kingdom; Research & Development, GSK, Philadelphia, PA, United States; Division of Pulmonary and Critical Care, Rutgers New Jersey Medical School, Newark, NJ, United States; Biostatistics, GSK, Bengaluru, Karnataka, India; Global Medical Affairs, GSK, London, United Kingdom; Nuffield Department of Clinical Medicine, University of Oxford, Oxford, United Kingdom

**Keywords:** biological therapy, noninferiority trial, anti-asthmatic agents

## Abstract

**Rationale:**

Depemokimab is the first ultra-long-acting biologic with high IL-5 binding affinity, high potency, and an extended half-life enabling twice-yearly dosing.

**Objectives:**

Investigate the efficacy and safety of switching to depemokimab in participants with severe asthma already managed with and responsive to short-acting biologic therapies targeting IL-5 or its receptor.

**Methods:**

NIMBLE (NCT04718389) was a multicenter, randomized, double-blind, double-dummy, parallel-group, phase 3A noninferiority study. Participants were ≥12 years old with asthma and documented clinical benefit on mepolizumab 100 mg subcutaneously every 4 weeks or benralizumab 30 mg subcutaneously every 8 weeks for ≥12 months. Participants were randomized 1:1 to depemokimab 100 mg subcutaneously every 26 weeks or maintained on their prior biologic (mepolizumab or benralizumab). The primary endpoint was annualized rate of clinically significant exacerbations over 52 weeks, with predefined noninferiority margin set at 1.28. Safety endpoints included adverse events.

**Measurements and Main Results:**

Annualized rates (95% confidence intervals [CIs]) of clinically significant exacerbations over 52 weeks were 0.57 (0.50 to 0.64) with depemokimab (*n* = 848) and 0.49 (0.43 to 0.55) with active comparator (*n* = 839); the rate ratio (95% CI) was 1.16 (0.98 to 1.38). Since the upper bound of the 95% CI exceeded 1.28, noninferiority was not met. Most participants in both treatment arms experienced no clinically significant exacerbations. Health-related quality of life, asthma control, and lung function outcomes were stable throughout the study. Adverse events were comparable between treatment groups.

**Conclusions:**

While statistical noninferiority was not met, exacerbation rates were low and symptom control/lung function were maintained in both groups. This first randomized, controlled switch trial in severe asthma suggests that participants with severe asthma on mepolizumab or benralizumab may safely switch to twice-yearly depemokimab.

## Graphical Abstract

**Figure:**
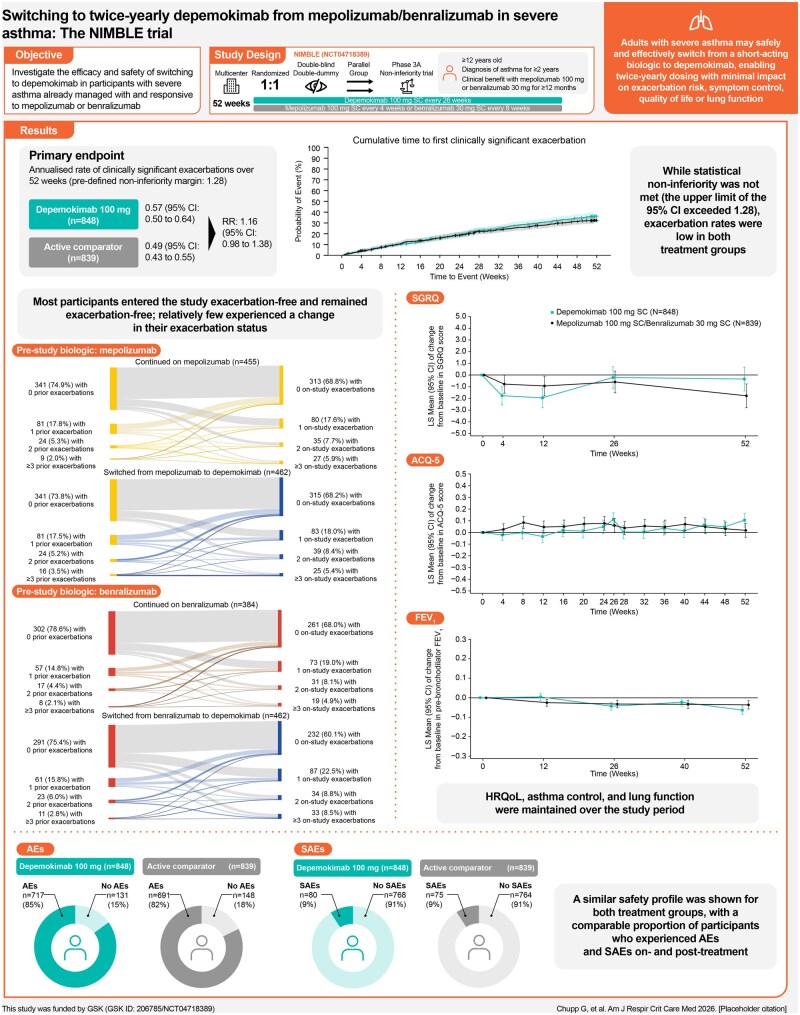

At a Glance Commentary **Scientific Knowledge on the Subject:** Prior evidence has shown the benefits of targeting IL-5 signaling to improve outcomes in severe asthma. Current biologic therapies targeting IL-5 or its receptor include mepolizumab and benralizumab, with dosing schedules every 4-8 weeks. Many patients prefer biologics with less frequent dosing intervals, which are associated with improved adherence and outcomes. Depemokimab is the first ultra-long-acting biologic with high IL-5 binding affinity, high potency, and an extended half-life enabling twice-yearly dosing, and has shown efficacy in type 2 asthma (in the SWIFT-1/-2 studies). Little is known about switching patients already benefiting from biologic therapy onto alternative treatments.

**What This Study Adds to the Field:** This is the first randomized controlled switch trial in severe asthma. The results showed that annualized exacerbation rates remained low when switching to depemokimab versus continuing mepolizumab/benralizumab treatment, with variation in exacerbation rates observed according to prestudy biologic treatment. Health-related quality of life, asthma control, and lung function measures were maintained throughout the study, with comparable safety in all treatment groups. Although the study did not meet the statistical noninferiority criterion for annualized exacerbation rates, the results suggest participants with severe asthma can safely and effectively be switched from a short-acting biologic to twice-yearly dosing with depemokimab.

## Introduction

Biologic therapy is a well-established therapy for severe asthma, leading to clinical disease improvement when added to moderate/high-dose inhaled corticosteroids (ICSs) and a second controller.^1-7^ These therapies target type 2 inflammation, which affects >80% of patients and is mediated mainly by IL-4, IL-5, and IL-13.^8^^,^^9^ While some patients may be able to safely discontinue biologic therapy, sustained treatment is needed to provide optimal long-term clinical benefits for many patients.^10^^,^^11^

Of the approved biologics targeting type 2 inflammation, 3 are directed against IL-5 (ie, mepolizumab, reslizumab) or its receptor (benralizumab) and are indicated for severe asthma with an eosinophilic phenotype.^12-14^ In addition to depleting proinflammatory eosinophils,^15^^,^^16^ therapies targeting the IL-5 pathway have also been shown to rebalance the immune response,^17^^,^^18^ facilitate clearance of mucus plugs,^19^ restore epithelial integrity,^20^ and reverse airway remodeling in patients with severe asthma.^21^^,^^22^ This broad biological impact is indicative of the key role of IL-5 in regulating eosinophils and a range of other cells involved in type 2 airway inflammation,^23^ resulting in the clinical benefits experienced with sustained long-term anti-IL-5 therapy in severe asthma.^24^

The long-term goal of asthma management is to reduce the risk of exacerbations, lung function decline, and systemic corticosteroid (SCS) use, in addition to achieving control of symptoms that impact patients’ daily lives.^1^ In real-world use, mepolizumab and benralizumab can reduce exacerbation rates and oral corticosteroid (OCS) burden (reduction and elimination of maintenance OCS use as well as decreased need for rescue bursts), improve disease control,^25^^,^^26^ and potentially prevent lung function decline^27^ in patients with severe asthma. However, there is an associated treatment burden of frequent dosing, as approved biologic therapies require dosing from every 2 to every 8 weeks.^28^ Biologics with longer dosing intervals are preferred by both patients and physicians, and may be associated with better adherence over therapies that require more frequent dosing.^29^^,^^30^ Critically, improved adherence is associated with better outcomes.^31^^,^^32^ As such, a biologic treatment option for severe asthma that supports adherence/persistence over time may help patients to attain maximum clinical benefit.

Depemokimab is the first ultra-long-acting biologic with high IL-5 binding affinity, high potency, and an extended half-life, enabling sustained suppression of type 2 inflammation and twice-yearly dosing in patients with asthma.^33^^,^^34^ Two replicate phase 3A, randomized, controlled studies (SWIFT-1 and SWIFT-2) demonstrated that depemokimab was associated with significantly greater reductions in the annualized rate of exacerbations versus placebo (by 54%) and exacerbations leading to hospitalization/emergency department (ED) visits (by 72%), with a similar frequency of adverse events (AEs).^34^ Additionally, these responses to depemokimab have been sustained with up to 2 years of exposure in AGILE, the long-term extension study of SWIFT-1 and SWIFT-2.^35^ It remains of clinical interest to assess the effectiveness of switching adults who have a long-standing benefit from short-acting biologics to twice-yearly depemokimab, which may provide sustained suppression of type 2 inflammation and allow long-term clinical benefits to be maintained.

The NIMBLE study was the first blinded, randomized controlled switch trial designed to address a clinically relevant data gap in the ability to switch biologics and maintain long-term disease control in people with severe asthma. Specifically, the study aimed to investigate whether switching to depemokimab (once every 26 weeks) is noninferior to maintaining current therapy in participants with asthma who have previously benefited from mepolizumab (every 4 weeks) or benralizumab (every 8 weeks).

## Methods

### Study design

NIMBLE was a multicenter, randomized, double-blind, double-dummy, parallel-group, phase 3A noninferiority study. The study was conducted in accordance with consensus ethical principles derived from international guidelines including the Declaration of Helsinki and Council for International Organizations of Medical Sciences international ethical guidelines, applicable International Conference on Harmonisation and Good Clinical Practice guidelines, and any additional applicable laws and regulations. The trial is complete and registered with ClinicalTrials.gov (NCT04718389; GSK ID: 206785).

Participants were randomized 1:1 into one of 2 treatment groups: (1) depemokimab 100 mg subcutaneously (SC) administered every 26 weeks and placebo SC matching the dosing schedule of the participant’s treatment prior to randomization (ie, mepolizumab every 4 weeks or benralizumab every 8 weeks), or (2) active comparator according to the participant’s treatment prior to randomization (ie, either mepolizumab every 4 weeks or benralizumab every 8 weeks) and placebo SC matching the depemokimab dosing schedule every 26 weeks (Figure S1). Randomization was stratified by prior biologic. All participants continued their baseline standard of care asthma therapy during the study. In the active comparator arm, a minimum of 40% of participants on each treatment were to be included. Full details of the randomization and blinding procedures are detailed in Appendix S1.

**Figure 1:**
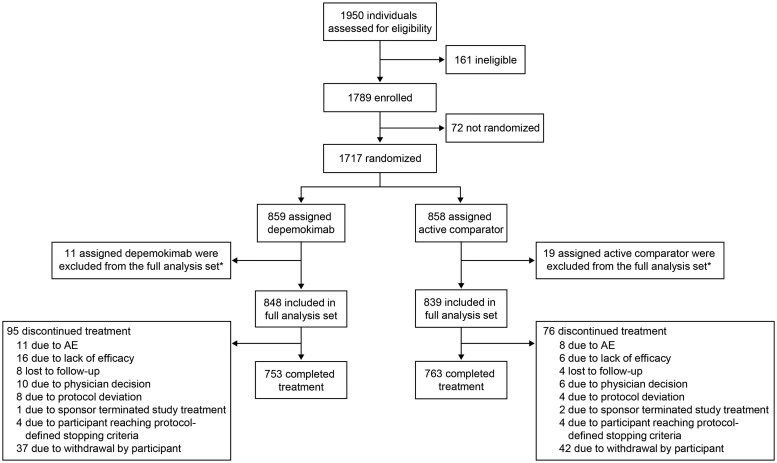
Trial profile for NIMBLE. *A total of 30 participants (ie, 11 participants assigned depemokimab and 19 participants assigned active comparator) were excluded from the full analysis set (27 due to concerns about data integrity and good clinical practice violations and 3 who did not receive study treatment). Abbreviation: AE, adverse event.

### Participants

Participants were recruited at study sites and provided written informed consent to participate in the study. Key eligibility criteria were: adults and adolescents (≥12 years old at time of consent); physician-documented diagnosis of asthma for ≥2 years; and receiving either mepolizumab 100 mg SC or benralizumab 30 mg SC for ≥12 months prior to screening with documented clinical benefit (by meeting one of the following 3 criteria based on investigator documentation: ≥50% reduction in exacerbation frequency; ≥50% reduction in maintenance OCS use since treatment initiation; no exacerbations in prior 6 months and an Asthma Control Questionnaire-5 [ACQ-5] score ≤1.5 at screening). Treatment with mepolizumab and benralizumab was initiated at the discretion of their treating physician in real-world settings, and no treatment history was collected beyond the prior 12 months. Patients who were receiving reslizumab were not eligible for the study due to the complications of blinding an intravenous medication that requires weight-based dosing. Full details of eligibility criteria and clinical benefit definitions are detailed in Appendix S1.

### Outcomes

The primary endpoint was the annualized rate of clinically significant exacerbations over 52 weeks. Clinically significant exacerbations were defined as worsening of asthma requiring SCS, and/or hospitalization, and/or ED visit. Prespecified secondary endpoints were weighted mean change (Appendix S1) from baseline in St. George’s Respiratory Questionnaire (SGRQ) total score, ACQ-5 score, and prebronchodilator forced expiratory volume in 1 second (FEV_1_), over 52 weeks. Other prespecified efficacy endpoints included the annualized rate of exacerbations requiring hospitalization and/or ED visit over 52 weeks, and time to first clinically significant exacerbation. Pharmacodynamics was assessed as ratio to baseline in absolute blood eosinophil count over 52 weeks. Safety endpoints included incidence of AEs, serious AEs (SAEs), AEs of special interest, incidence of immunogenicity (as measured by presence of anti-drug or neutralizing antibodies to depemokimab), and assessment of vital signs, electrocardiogram values, and laboratory parameters.

### Statistical analysis

All efficacy and safety endpoints were evaluated in the full analysis set, comprising all participants who received at least one dose of study intervention, but excluding participants from sites with Good Clinical Practice noncompliance or significant data integrity concerns.

A sample size of 850 participants in each of the 2 study arms (ie, depemokimab and combined mepolizumab/benralizumab) was calculated to achieve sufficient power to conclude noninferiority for the primary endpoint, with a margin of 1.28, using a one-sided 2.5% significance level. Noninferiority was met if the upper bound of the 95% confidence interval (CI) was <1.28. The choice of noninferiority margin was based on the application of the fixed margin approach. A meta-analysis of anti-IL-5 therapies compared with placebo was conducted to estimate the treatment effect (exacerbation rate ratio [RR]) of the active comparator group. Based on the assumption that this treatment effect would be similar in NIMBLE, the exacerbation RR for depemokimab that would result in placebo-like efficacy compared with the active comparator group was subsequently estimated. The noninferiority margin specified above was selected as it preserved 50% of the active comparator treatment effect on the log_e_ scale. Full statistical details are provided in Appendix S1.

The primary endpoint (annualized rate of exacerbations) was analyzed using a negative binomial model, including several covariates as detailed in Appendix S1. RRs and 95% CIs were calculated to compare depemokimab and the active comparator group (mepolizumab/benralizumab). Both primary and supplementary estimands were conducted for the primary endpoint; full details are provided in Appendix S1. Secondary endpoints were analyzed using an analysis of covariance model, including covariates as detailed in Appendix S1. Differences in the means and 95% CIs were calculated for the comparison between treatment arms. Of the other endpoints, the annualized rate of exacerbations requiring hospitalization and/or ED visit was analyzed using the same approach as the primary endpoint. The proportion of participants who experienced no clinically significant exacerbations is also reported. Absolute and ratio to baseline in blood eosinophil counts were analyzed using summary statistics. A prespecified subgroup analysis was conducted to assess the effect of depemokimab on primary and secondary endpoints, according to prestudy biologic treatment for asthma.

Post hoc analyses were performed to assess the distribution of number of clinically significant exacerbations experienced during the study according to prestudy biologic and number of exacerbations in the prior 12 months. Full details of the analysis, including determination of the noninferiority margin and the intercurrent events strategy for each efficacy endpoint, are listed in Appendix S1.

## Results

From January 26, 2021 to August 16, 2024, 1950 individuals were screened for NIMBLE and 1717 were randomized, with 30 subsequently excluded. The remaining 1687 participants comprised the full analysis set (848 received depemokimab; 839 received active comparator) (Figure 1). The proportion of participants meeting each of the eligibility criteria for documented clinical benefit with mepolizumab or benralizumab were similar between treatment groups (Table S1). Baseline demographics and clinical characteristics were similar between treatment groups (Table 1). The mean age of all participants was 58.6 years (standard deviation [SD], 13.2 years), with 22.4 years (SD, 16.5 years) mean duration of asthma. The mean number of exacerbations in the 12 months prior to enrollment in NIMBLE was 0.41 in the depemokimab group and 0.34 in the active comparator group (values derived post hoc). Most participants had no exacerbations requiring SCS in the prior 12 months (75% in the depemokimab group and 77% in the active comparator group). In both the depemokimab and active comparator groups, 97% of participants had no exacerbations requiring hospitalization in the prior 12 months.

**Table 1:** Baseline demographics and clinical characteristics.

Characteristic	**Depemokimab (*n*** = **848)**	**Active comparator (mepolizumab/benralizumab) (*n*** = **839)**
**Age, y, mean (SD)**	58.3 (13.2)	58.9 (13.2)
**Female sex, No. (%)**	528 (62)	522 (62)
**Race, No. (%)**	843	832
** Asian**	148 (18)	140 (17)
** Black or African American**	26 (3)	35 (4)
** White**	659 (78)	645 (78)
** Other^a^**	10 (1)	12 (1)
**Region, No. (%)**		
** Europe**	410 (48)	407 (49)
** United States**	220 (26)	214 (26)
** Rest of the world**	218 (26)	218 (26)
**Duration of asthma, y, mean (SD)**	22.0 (16.1)	22.7 (16.9)
**BEC, No.**	835	830
** Geometric mean, cells/µL (95% CI)**	30 (28-33)	29 (26-31)
**Prebronchodilator FEV_1_, No.**	814	803
** L, mean (SD)**	2.2 (0.8)	2.2 (0.8)
**Percent predicted FEV_1_, No.**	814	803
** %, mean**	80.7	80.6
**ICS use, No. (%)**		
** Medium dose**	421 (50)	409 (49)
** High dose**	427 (50)	430 (51)
**LAMA, No. (%)**		
** Yes**	408 (48)	384 (46)
** No**	440 (52)	455 (54)
**Maintenance OCS, No. (%)**		
** Yes**	36 (4)	28 (3)
** No**	812 (96)	811 (97)
**Baseline OCS daily dose (prednisone equivalent), No. (%)**	36 (4)	28 (3)
** <7.5 mg/d**	25 (69)	20 (71)
** ≥7.5 to <15 mg/d**	8 (22)	6 (21)
** ≥15 to <30 mg/d**	1 (3)	2 (7)
** ≥30 mg/d**	2 (6)	0
**No. of exacerbations (prior 12 mo), mean (SD)**	0.41 (0.99)	0.34 (0.77)
**No. of exacerbations requiring OCS/SCS in prior 12 mo, No. (%)**		
** 0**	632 (75)	643 (77)
** 1**	142 (17)	138 (16)
** 2**	47 (6)	41 (5)
** 3**	16 (2)	10 (1)
** 4**	6 (<1)	4 (<1)
** >4**	5 (<1)	3 (<1)
**No. of exacerbations requiring hospitalization in prior 12 mo, No. (%)**		
** 0**	826 (97)	814 (97)
** 1**	18 (2)	20 (2)
** 2**	2 (<1)	5 (<1)
** 3**	2 (<1)	0
** 4**	0	0
** >4**	0	0
**Nasal polyps, No. (%)**	156 (18)	142 (17)

Abbreviations: BEC, blood eosinophil count; CI, confidence interval; FEV_1_, forced expiratory volume in 1 second; ICS, inhaled corticosteroid; LAMA, long-acting muscarinic antagonist; OCS, oral corticosteroid; SCS, systemic corticosteroid; SD, standard deviation.

a American Indian or Alaska Native, Native Hawaiian or other Pacific Islander, or mixed race.

The annualized rate of clinically significant exacerbations over 52 weeks (primary endpoint) was 0.57 (95% CI, 0.50 to 0.64) with depemokimab and 0.49 (95% CI, 0.43 to 0.55) with active comparator (RR, 1.16 [95% CI, 0.98 to 1.38]). Therefore, the study did not meet the statistical criterion of noninferiority as the upper limit of the 95% CI exceeded the predefined margin of 1.28 (Table 2). The supplementary estimand for the primary endpoint (which excluded participants who discontinued for any reason or used prohibited medication) was consistent with the primary analysis (RR, 1.13 [95% CI, 0.94 to 1.36]). In the prespecified subgroup analysis by prestudy biologic treatment (Table 3), for participants previously on mepolizumab, the rates of clinically significant exacerbations were the same (0.48) with comparable 95% CIs for both treatments (0.40 to 0.57 for depemokimab; 0.41 to 0.58 for mepolizumab). For participants who continued benralizumab, the rate of clinically significant exacerbations was also 0.48 (95% CI, 0.41 to 0.58). However, the rate was higher in participants who switched from benralizumab to depemokimab (0.67 [95% CI, 0.57 to 0.79]).

**Table 2:** Summary of primary and secondary endpoints.

Endpoint	**Depemokimab (*n*** = **848)**	**Active comparator (mepolizumab/benralizumab) (*n*** = **839)**
**Primary endpoint: annualized rate of clinically significant exacerbations over 52 weeks**		
** No.**	813	803
** Annualized rate (95% CI)**	0.57 (0.50 to 0.64)	0.49 (0.43 to 0.55)
** RR (95% CI)**	1.16 (0.98 to 1.38)	
**Primary endpoint (supplementary estimand): annualized rate of clinically significant exacerbations over 52 weeks**		
** No.**	775	757
** Annualized rate (95% CI)**	0.51 (0.45 to 0.58)	0.45 (0.39 to 0.52)
** RR (95% CI)**	1.13 (0.94 to 1.36)	
**Secondary endpoint: weighted mean change from baseline to week 52 in SGRQ total score**		
** No.**	765	755
** LS mean (SE)**	25.94 (0.48)	25.76 (0.49)
** LS mean change (SE)**	-0.69 (0.48)	-0.86 (0.49)
** Treatment difference (95% CI)**	0.17 (-0.85 to 1.20)	
**Secondary endpoint: weighted mean change from baseline to week 52 in ACQ-5**		
** No.**	773	758
** LS mean (SE)**	0.94 (0.03)	0.97 (0.03)
** LS mean change (SE)**	0.03 (0.03)	0.06 (0.03)
** Treatment difference (95% CI)**	-0.03 (-0.09 to 0.02)	
**Secondary endpoint: weighted mean change from baseline to week 52 in FEV_1_**		
** No.**	795	782
** LS mean (SE)**	2.20 (0.009)	2.19 (0.010)
** LS mean change (SE)**	-0.014 (0.009)	-0.018 (0.010)
** Treatment difference (95% CI)**	0.004 (-0.016 to 0.024)	

Abbreviations: ACQ-5, Asthma Control Questionnaire-5; CI, confidence interval; FEV_1_, forced expiratory volume in 1 second; LS, least squares; RR, rate ratio; SE, standard error; SGRQ, St. George’s Respiratory Questionnaire.

**Table 3:** Prespecified subgroup analysis of primary and secondary endpoint according to prestudy biologic treatment.

Endpoint	Prestudy mepolizumab		Prestudy benralizumab	
	Depemokimab	Active comparator	Depemokimab	Active comparator
**Primary endpoint: annualized rate of clinically significant exacerbations over 52 weeks**				
** No.**	442	432	371	371
** Annualized rate (95% CI)**	0.48 (0.40 to 0.57)	0.48 (0.41 to 0.58)	0.67 (0.57 to 0.79)	0.48 (0.41 to 0.58)
** RR (95% CI)**	0.99 (0.78 to 1.26)		1.38 (1.09 to 1.75)	
**Secondary endpoint: weighted mean change from baseline to week 52 in SGRQ total score**				
** No.**	416	407	349	348
** LS mean (SE)**	25.19 (0.63)	26.12 (0.63)	26.62 (0.73)	25.09 (0.77)
** LS mean change (SE)**	-0.98 (0.63)	-0.05 (0.63)	-0.53 (0.73)	-2.06 (0.77)
** Treatment difference (95% CI)**	-0.93 (-2.29 to 0.43)		1.53 (-0.02 to 3.09)	
**Secondary endpoint: weighted mean change from baseline to week 52 in ACQ-5**				
** No.**	420	409	353	349
** LS mean (SE)**	0.92 (0.03)	0.99 (0.03)	0.96 (0.04)	0.94 (0.04)
** LS mean change (SE)**	0.01 (0.03)	0.08 (0.03)	0.04 (0.04)	0.03 (0.04)
** Treatment difference (95% CI)**	-0.07 (-0.14 to 0.00)		0.02 (-0.07 to 0.10)	
**Secondary endpoint: weighted mean change from baseline to week 52 in FEV_1_**				
** No.**	433	423	362	359
** LS mean (SE)**	2.22 (0.012)	2.19 (0.012)	2.18 (0.014)	2.21 (0.015)
** LS mean change (SE)**	-0.017 (0.012)	-0.047 (0.012)	-0.006 (0.014)	0.024 (0.015)
** Treatment difference (95% CI)**	0.030 (0.003 to 0.056)		-0.030 (-0.060 to 0.001)	

Abbreviations: ACQ-5, Asthma Control Questionnaire-5; CI, confidence interval; FEV_1_, forced expiratory volume in 1 second; LS, least squares; RR, rate ratio; SE, standard error; SGRQ, St. George’s Respiratory Questionnaire.

**Figure 2:**
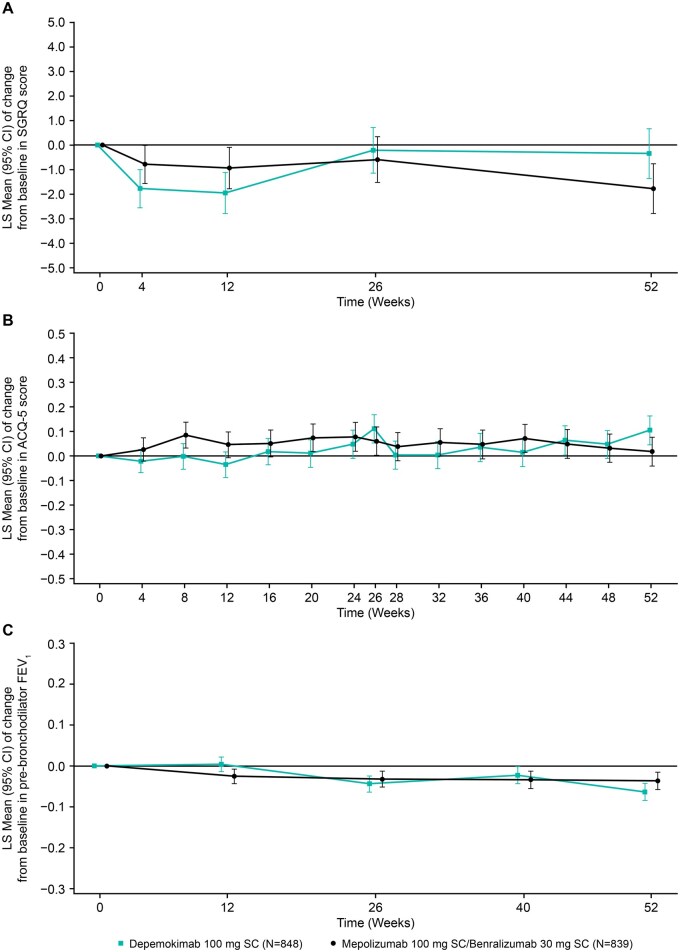
LS Mean changes from baseline calculated over 52 weeks, in SGRQ total score (A), ACQ-5 score (B), and FEV_1_ (C). The number of participants contributing to the analyses was 765 (depemokimab) and 755 (mepolizumab/benralizumab) for SGRQ, 773 (depemokimab) and 758 (mepolizumab/benralizumab) for ACQ-5, and 795 (depemokimab) and 782 (mepolizumab/benralizumab) for FEV_1_ (all values at baseline). Abbreviations: ACQ-5, Asthma Control Questionnaire-5; CI, confidence interval; FEV_1_, forced expiratory volume in 1 second; LS, least squares; SC, subcutaneous; SGRQ, St. George’s Respiratory Questionnaire.

For the secondary endpoints, SGRQ total scores remained relatively stable (Figure 2A), with a comparable weighted mean change (standard error) from baseline over 52 weeks with depemokimab versus active comparator and a treatment difference of 0.17 (95% CI, -0.85 to 1.20) (Table 2). ACQ-5 scores were also stable with minimal changes from baseline over 52 weeks in both treatment groups throughout the study (Figure 2B), with a treatment difference of -0.03 (95% CI, -0.09 to 0.02) (Table 2). Similarly, prebronchodilator FEV_1_ (L) remained stable in both treatment groups during the study (Figure 2C), with a treatment difference of 0.004 (95% CI, -0.016 to 0.024) (Table 2). In the prespecified subgroup analysis (Table 3), there were minimal changes in weighted mean SGRQ total score across all treatment groups. Weighted mean change in ACQ-5 score and FEV_1_ with depemokimab over 52 weeks was comparable to that observed in the groups continuing mepolizumab or benralizumab.

**Figure 3:**
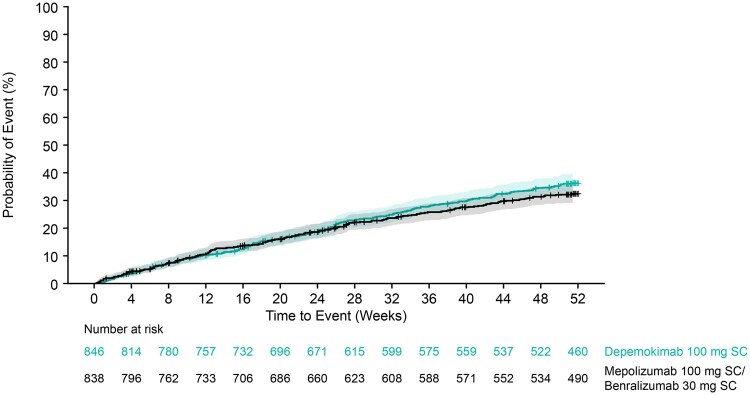
Kaplan–Meier curve for cumulative time to first clinically significant exacerbation. Abbreviation: SC, subcutaneous.

**Figure 4:**
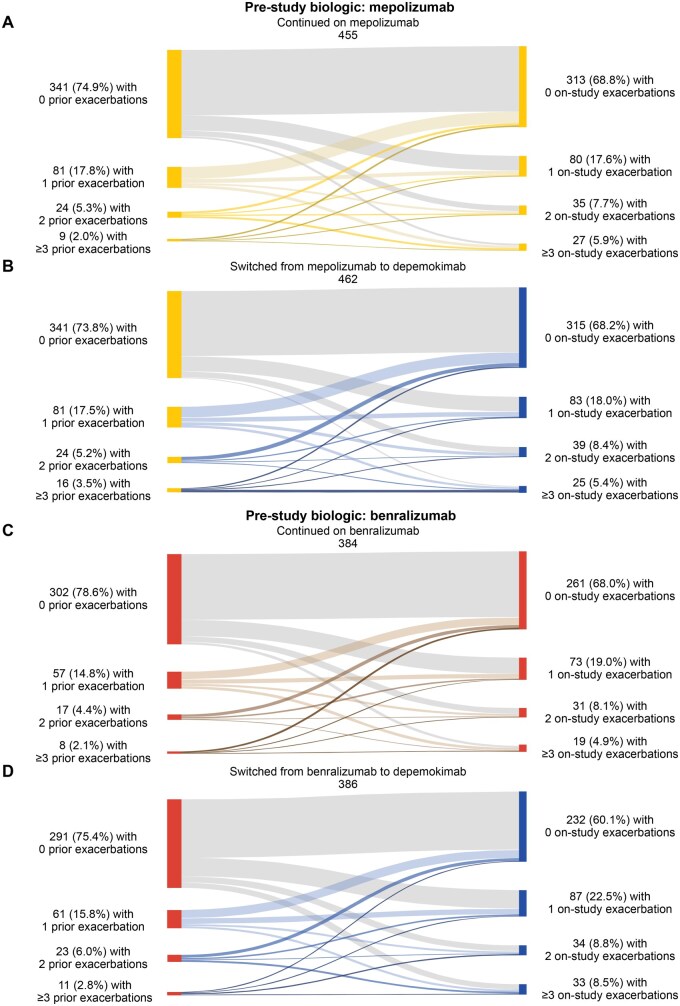
(A-D) Numbers of clinically significant exacerbations experienced in the 12 months prior to the study and during the study, by prestudy biologic and on-study treatment.

The results observed for the other efficacy endpoints were comparable between treatment groups. The annualized rate of exacerbations requiring hospitalization and/or ED visit was 0.06 (95% CI, 0.05 to 0.09) with depemokimab and 0.06 (95% CI, 0.04 to 0.08) with active comparator (RR, 1.12 [95% CI, 0.70 to 1.78]). Analysis of the time to first clinically significant exacerbation showed that the probability of patients experiencing an event at any time over the 52 weeks was 36% (95% CI, 33% to 40%) with depemokimab and 32% (95% CI, 29% to 36%) with active comparator (hazard ratio, 1.14 [95% CI, 0.96 to 1.34]). There was a trend toward a higher probability of experiencing a clinically significant exacerbation with depemokimab at timepoints beyond 24 weeks, although CIs did overlap throughout the study (Figure 3). Most participants who did not experience any exacerbations in the 12 months prior to the study continued to have no clinically significant exacerbations during the study (Figure 4). Similar patterns in the number of clinically significant exacerbations experienced prior to and during the study were observed regardless of treatment group. The proportion of participants without any clinically significant exacerbation was 64% with depemokimab and 68% with active comparator. In participants previously receiving mepolizumab, the proportion without any clinically significant exacerbations was the same (68%), regardless of whether they were switched to depemokimab or not. In participants previously receiving benralizumab, the proportion without any clinically significant exacerbations was 59% in those switching to depemokimab and 68% in those remaining on benralizumab. Most participants did not experience exacerbations requiring hospitalization and/or ED visit (94% with depemokimab, 95% with active comparator).

At baseline, geometric mean blood eosinophil count was 12 cells/µL in both groups of participants previously treated with benralizumab (ie, those who continued on benralizumab and those who switched to depemokimab). The corresponding values in participants previously treated with mepolizumab were 60 cells/µL and 63 cells/µL in participants continuing mepolizumab and those who switched from mepolizumab to depemokimab, respectively. In participants previously treated with benralizumab who switched to depemokimab, blood eosinophil count was initially sustained up to week 12, increased to 45 cells/µL at week 26, and then remained relatively stable. Blood eosinophil counts were maintained at approximately baseline levels in participants who continued benralizumab. In participants previously treated with mepolizumab, blood eosinophil count remained relatively stable in both the group that switched to depemokimab and the group that continued mepolizumab. In participants previously treated with mepolizumab who switched to depemokimab, blood eosinophil count decreased slightly between weeks 4 and 20 but rose to baseline level at week 26; this pattern was repeated between weeks 26 and 52. Changes in blood eosinophil counts by prestudy biologic are shown in Figure S2.

The proportion of participants who experienced AEs on treatment and posttreatment was similar in the depemokimab (85%) and active comparator (82%) groups (Table 4). SAEs were reported in 9% of participants in both treatment groups. In the active comparator group, there were 3 (<1%) SAEs considered related to study treatment by the investigator, and 1 fatal SAE that was not considered related to study treatment by the investigator (no treatment-related or fatal SAEs occurred in participants receiving depemokimab). Anti-drug antibodies were observed in 4% of participants receiving depemokimab at any time during the study period, and 2 participants receiving depemokimab had neutralizing antibodies. Assessment of binding or neutralizing antibodies was not performed in participants receiving active comparator at any point postbaseline.

**Table 4:** Summary of adverse events in the safety population on treatment and posttreatment.

Event	**Depemokimab (*n*** = **848)**	**Active comparator (*n*** = **839)**
**AEs, No. (%)**		
** Any AE**	717 (85)	691 (82)
** Related to study treatment**	78 (9)	82 (10)
** Leading to permanent discontinuation of study treatment or study withdrawal**	13 (2)	12 (1)
** Leading to dose interruption or delay**	11 (1)	22 (3)
**SAEs, No. (%)**		
** Any SAE**	80 (9)	75 (9)
** Related to study treatment**	0	3 (<1)
** Fatal**	0	1 (<1)
** Fatal, related to study treatment**	0	0

Abbreviations: AE, adverse event; SAE, serious adverse event.

## Discussion

The NIMBLE phase 3A study was the first randomized, controlled switch study in asthma, assessing 3 different biologics; the study investigated the efficacy and safety of switching participants with severe asthma established on and responsive to mepolizumab every 4 weeks or benralizumab every 8 weeks to twice-yearly depemokimab administered once every 26 weeks. While the primary endpoint did not meet the statistical criterion of noninferiority, the annualized rate of clinically significant exacerbations was low in both the depemokimab and active comparator groups. Furthermore, SGRQ/ACQ-5 scores and FEV_1_ remained relatively stable over the study period, suggesting that it is feasible to switch patients from mepolizumab or benralizumab to depemokimab and still maintain asthma control. Depemokimab presented a similar safety profile to mepolizumab/benralizumab, consistent with previously published results.^2^^,^^6^^,^^34^

A prespecified subgroup analysis showed that for participants previously on mepolizumab, annualized rates of clinically significant exacerbations were the same regardless of whether they were switched to depemokimab or remained on mepolizumab. For participants previously on benralizumab, the exacerbation rate was higher in those who switched to depemokimab compared with those who remained on benralizumab. This slight increase in exacerbation rate suggests that occasionally patients may not do as well switching to depemokimab, but the likelihood of exacerbation is low and not clinically meaningful given the low overall exacerbation rates in the year prior to NIMBLE (0.3–0.4/year, translating to a likelihood of one exacerbation every 2–3 years). For the overall group of participants switching to depemokimab, the exacerbation rate increased by 0.08 of an exacerbation per year relative to participants continuing with active comparator, equating to one additional exacerbation every 12.5 years. In participants switching from benralizumab to depemokimab, the exacerbation rate increased by 0.19 of an exacerbation per year, equating to one additional exacerbation every 5.3 years. Furthermore, most participants experienced no clinically significant exacerbations and nearly all avoided exacerbations leading to hospitalization during the study. The Sankey plot (Figure 4) shows that most participants entered the study exacerbation-free and remained exacerbation-free, with relatively few experiencing a change in their exacerbation status.

The difference observed between treatment groups appears to have been driven by a small subset of participants switching from benralizumab to depemokimab; there are several potential factors that may have contributed to this difference. All participants initiated mepolizumab or benralizumab treatment in a real-world setting, at least 1 year prior to enrollment in NIMBLE. It is possible that differences in participant characteristics at the time of biologic initiation might have led to differences between the populations due to variations in patient selection by treating physicians. Notably, while the statistical model adjusted for baseline exacerbation rates, the higher rate observed in the depemokimab versus active comparator group does indicate potential differences in characteristics pre-NIMBLE. Also, it is not known if the benralizumab population included participants who had experienced previous treatment failure with mepolizumab. The XALOC-1 real-world study of patients receiving benralizumab indicated that 62% of biologic-experienced patients had received mepolizumab; most had discontinued due to lack of efficacy and experienced smaller improvements in outcomes compared with biologic-naive patients.^36^ This observation may have played a role in NIMBLE, since depemokimab targets IL-5 similarly to mepolizumab. It is also important to consider the relationship between biologic therapies and ICS use. Real-world evidence has shown that lower ICS adherence appears to be relatively well tolerated in patients receiving benralizumab.^37^ In comparison, lesser reductions in OCS use and exacerbation rates have been observed in OCS-dependent patients initiated on mepolizumab.^38^ Since ICS adherence was not objectively monitored in NIMBLE, this cannot be investigated further.

In NIMBLE, blood eosinophil count increased in participants who switched from benralizumab to depemokimab, consistent with the known differences in mode of action between study treatments.^15^^,^^39^ However, most participants experienced no exacerbations despite showing an increase in blood eosinophil count, suggesting that this characteristic alone does not dictate clinical effect (as supported by the results from the ORACLE study).^40^ It is thus possible that participants initiating benralizumab are intrinsically different to those initiating mepolizumab and so have a lower threshold for response to an absolute blood eosinophil count, which becomes evident when switching to depemokimab. Alternatively, prolonged airway depletion of eosinophils with benralizumab may have altered the airway microenvironment,^41^^,^^42^ leading to the same altered threshold for response. Neither of these hypotheses can be assessed based on the available NIMBLE data but may be elucidated in future studies.

Participants in the NIMBLE study were distinct from trial populations previously studied in the randomized trials and real-world studies of mepolizumab, benralizumab, and depemokimab (which usually have included participants initiating biologic treatment). NIMBLE participants were already established on a biologic and assessed as responders; 75% and 77% of participants in the depemokimab and active comparator groups, respectively, had experienced no exacerbations requiring OCS/SCS at baseline. Therefore, the NIMBLE study does not inform about relative biologic effectiveness at biologic initiation, but rather provides evidence for outcomes when undertaking a switch from a short-acting biologic to an ultra-long-acting one.

Patients and physicians are known to prefer biologic therapies with longer dosing intervals,^29^^,^^30^ and these therapies can improve adherence^30^^,^^32^ with associated improvements in outcomes,^31^^,^^32^ as well as reducing the treatment burden on individuals and healthcare resources.^43^ Biologic switching is currently variable in real-world practice^36^^,^^44^^,^^45^ but may be on the increase with the availability of more treatment options for severe asthma^46^ and may provide clinical benefits.^45^^,^^47^ Clinicians and patients may use the results described here to inform conversations about switching and to weigh the importance of benefits (ie, similar control of symptoms, quality of life, and lung function, alongside a lower number of annual SC injections) versus risks (ie, a potential small increased risk of exacerbation in a subset of patients). In particular, although the increased risk of exacerbations is small, it raises the potential for increased long-term OCS exposure. However, it is important to note that a key predictor of outcome is treatment persistence, and that 40%–50% of patients in real-world studies were nonpersistent at 1 year following biologic therapy initiation.^32^^,^^48^ Based on this, any potential risk of increased OCS exposure could be mitigated by better treatment adherence/persistence and the resulting improvements in clinical outcomes associated with longer dosing intervals. Other study strengths include its global, multicenter nature, which ensures that the results apply to a diverse population of patients with asthma, and the fact that all participants received active treatment.

Study limitations reflect the real-world setting in which participants were initiated on biologic treatment. Specifically, treatment history beyond the year prior to NIMBLE was not available, including information on duration of biologic use, decisions leading to mepolizumab or benralizumab treatment, and details of other treatments used (including prior history of biologic treatment and discontinuations). Therefore, any differences between the mepolizumab and benralizumab groups could not be accounted for in the analyses, and direct comparisons cannot be made between these groups. Additionally, beyond eosinophil counts, other markers of type 2 inflammation such as fractional exhaled nitric oxide were not captured, limiting the ability to characterize the inflammatory nature of exacerbations and interpretation of the results in the context of different mechanisms of action. Finally, as NIMBLE represents the first randomized switch study of biologics for asthma, the noninferiority margin was determined based on assumptions of treatment effect in placebo-controlled trials. While this approach was necessary given the lack of previous switch studies, the study population in NIMBLE differed substantially from historical studies, including participants who were well-controlled at study entry with low rates of exacerbations. Therefore, this difference may have impacted the assumptions on treatment effect. Consideration of such limitations will help inform the design of future biologic switching studies.

In conclusion, the results suggest that people with severe asthma and documented benefit from IL-5–directed biologic treatment can safely and effectively switch from a short-acting biologic such as mepolizumab or benralizumab to an ultra-long-acting biologic such as depemokimab. NIMBLE shows that this switch can be implemented with maintenance of disease control, preserved lung function, and no impairment of quality of life and with no adverse safety signals, underscoring the potential of depemokimab to transform asthma treatment.

## Supplementary Material

aamag031_Supplementary_Data

## Data Availability

The study sponsor will assess requests from qualified researchers for anonymized individual participant-level data and related study documents. Data sharing is subject to specific criteria, conditions, and exceptions. For further information, please refer to the GSK weblink to access GSK’s data sharing policies and, as applicable, seek anonymized subject-level data: https://www.gsk-studyregister.com/en/. This article has an online data supplement, which is accessible at the Supplements tab.
