# Supplementary material for: Switching to twice-yearly depemokimab from mepolizumab/benralizumab in severe asthma: a multicenter, randomized, double-blind, phase 3A clinical trial (NIMBLE)
Source: Am J Respir Crit Care Med. 2026 Feb 11;212(5):921–35. doi: 10.1093/ajrccm/aamag031 (PMC13160935; doi:10.1093/ajrccm/aamag031)
Supplement: Supplementary file 1 [file aamag031_supplementary_data.zip › NIMBLE_primary_ms_SUPPLEMENT_AJRCCM_Revision_1_CLEAN.docx]

**Switching to twice-yearly depemokimab from mepolizumab/benralizumab in severe asthma: A multicenter, randomized, double-blind, Phase 3A Clinical Trial (NIMBLE)**

Geoffrey Chupp, MD; Hiroyuki Nagase, PhD; Dirk Skowasch, PhD; Gilles Devouassoux, MD, PhD; Andréanne Côté, MD, MSc; Daniel J Jackson, MD; David J Jackson, PhD; Michael E Wechsler, MD; Varsha Imber, MSc; John E McGinniss, MD, MSTR; Sherine O K, MSc; Peter Howarth, MD; Ian D Pavord, FMedSci on behalf of the NIMBLE study investigators

# Online Data Supplement

## Supplementary figures and tables

### **Figure E1.** NIMBLE study design.

### **Figure E2.** Change in blood eosinophil count from baseline over 52 weeks, according to pre-study biologic

SC, subcutaneous

### **Table E1**. Proportions of participants meeting the eligibility criteria for documented clinical benefit of therapy with mepolizumab or benralizumab

| **n (%)*** | **Depemokimab**  **N=848** | **Active comparator**  **N=839** |
| --- | --- | --- |
| ≥50% reduction in exacerbation frequency since treatment initiation | 500 (59) | 492 (59) |
| ≥50% reduction in maintenance OCS use since treatment initiation | 201 (24) | 196 (23) |
| No exacerbations in prior 6 months and an ACQ-5 score ≤1.5 at screening | 470 (55) | 466 (56) |

*Participants could meet more than 1 criterion
ACQ-5, asthma control questionnaire-5; OCS, oral corticosteroid

## Appendix 1

### Study locations

NIMBLE was conducted in 390 centres, in 20 countries/regions (Australia, Austria, Canada, Finland, France, Germany, Ireland, Israel, Italy, Japan, Netherlands, Norway, Portugal, Slovenia, Spain, Sweden, Switzerland, Taiwan, UK, US)

### Eligibility criteria

Participants were recruited at study sites and provided written informed consent to participate in the study. Key eligibility criteria were: adults and adolescents (≥12 years old at time of consent); physician-documented diagnosis of asthma for ≥2 years; receiving either mepolizumab 100 mg subcutaneously (SC) or benralizumab 30 mg SC for ≥12 months prior to screening with documented clinical benefit (by meeting one of the following 3 criteria based on investigator documentation: ≥50% reduction in exacerbation frequency; ≥50% reduction in maintenance OCS use since treatment initiation; no exacerbations in prior 6 months and an asthma control questionnaire-5 [ACQ-5] score ≤1.5 at screening). Treatment with mepolizumab and benralizumab was initiated in real-world settings, and no treatment history was collected beyond the prior 12 months. Participants were required to have a documented need for regular treatment with medium to high dose ICS in the 12 months prior to Visit 1, with or without maintenance OCS (participants treated with medium dose ICS also required treatment with a long-acting beta-2-agonist [LABA]); and current treatment with ≥1 additional controller medication besides ICS. Participants with concurrent, clinically important respiratory diseases other than asthma (e.g. chronic obstructive pulmonary disease), or other eosinophilic diseases (e.g. eosinophilic granulomatosis with polyangiitis), were excluded.

*Primary endpoint: definition*

Clinically significant exacerbations were defined as worsening of asthma which requires use of systemic CS and/or hospitalization and/or Emergency Department (ED) visit. For all participants, IV or oral steroids (e.g., prednisone) for ≥3 days or a single IM CS dose was required. For participants on maintenance systemic CSs, at least double the existing maintenance dose for ≥3 days is required.

Exacerbations separated by ≤7 days will be treated as a continuation of the same exacerbation.

### Secondary endpoints: derivation of weighted mean

Weighted mean was calculated using the linear trapezoidal rule. The weighted mean was calculated as the AUC_(_*_tf – tl_*_)_ divided by the *tf- tl* weeks’ time interval for each participant (*tf* = time of first observation, *tl* = time for last observation).
where,

*Ci* = Change from baseline value at timepoint *I*

*ti* = Time in weeks for planned relative time point *i*

*i* = planned relative time

*f* = first *I*

*l* = last *I*

If an observation is missing between two non-missing observations (non-monotone missingness), the AUC was linearly interpolated between the two non-missing values i.e., the participant’s profile was assumed to be linear between the two available values.

### Randomization and masking

Participants were randomized 1:1 into one of two treatment groups: 1) depemokimab 100 mg SC administered every 26 weeks and placebo SC matching the dosing schedule of the participant’s treatment prior to randomization (i.e. mepolizumab every 4 weeks or benralizumab every 8 weeks), or 2) active comparator according to the participant’s treatment prior to randomization (i.e. either mepolizumab every 4 weeks or benralizumab every 8 weeks) and placebo SC matching depemokimab dosing schedule every 26 weeks. In the active comparator arm, a minimum of 40% of participants on each treatment were to be included. Treatment was assigned randomly using an interactive response technology system, with the randomization schedule generated using sponsor-validated software (RandALL NG) and was stratified by biologic received prior to the study (mepolizumab or benralizumab). Participants, investigators, and study site staff remained blinded to the study intervention throughout the study. To maintain the blind, all study interventions were administered by a prefilled syringe. Absolute and differential values of white blood cell counts (eosinophils, lymphocytes, basophils, neutrophils, and monocytes) from post-randomization samples were not reported to the site or central study teams, however total white blood cell counts were sent to sites throughout the study.

Depemokimab, mepolizumab, benralizumab, and placebo were all administered by the investigator or study site staff. Participants were monitored in the clinic for at least 2 hours after the first dose and Week 26 dose of depemokimab, for immediate hypersensitivity and any other reactions.

### Statistical analysis

The sample size of 850 participants per arm (equal allocation) is based on sufficient power to conclude non-inferiority with a margin of 1.28 using a one-sided 2.5% significance level. Should non-inferiority be met, the primary endpoint would be tested for superiority at the 5% 2-sided significance level.

The assumption of 0.8 for the dispersion parameter was observed in 2 mepolizumab phase 3 studies [Pavord, 2012; Ortega, 2014]. The assumption of 0.7 for the annual exacerbation rate for depemokimab and the active comparator arm was estimated from 2 mepolizumab open-label extension studies [Khatri, 2019; Lugogo, 2016] when taking into account the inclusion criteria for this study. Missing/excluded data is assumed at 10% of participant-years data (proportion of missing data observed in the first year of a mepolizumab open-label extension study [Lugogo, 2016]). An exacerbation rate ratio of 1 is assumed (depemokimab and comparator therapies are equally efficacious). Based on these assumptions, the power of the study is 91% [PASS, 2020]. Based on the assumptions above, the maximum treatment effect (i.e., observed rate ratio of depemokimab + SoC compared to active comparator (mepolizumab or benralizumab) + SoC estimated to result in a conclusion of non-inferiority is 1.11.

The choice of non-inferiority margin is based on the application of the fixed margin approach (Non-Inferiority Clinical Trials to Establish Effectiveness) [FDA, 2016]. The active control treatment effect was estimated by conducting a random-effects meta-analysis of competitor anti-IL-5 therapies (mepolizumab, benralizumab, reslizumab) compared with placebo. The rate ratio was estimated as 0.51 (95% CI: 0.42, 0.61). Based on an assumption of constancy (the effect of the active control compared with placebo would be similar in this study to that observed in the historical studies), the rate ratio for depemokimab compared with the active comparator arm (mepolizumab or benralizumab) that would result in a placebo-like efficacy for depemokimab is 1/0.61 = 1.64. The upper bound is used because this represents a conservative estimate of the effect the active comparator (mepolizumab, benralizumab) is expected to have. A non-inferiority margin of 1.28 preserves 50% of the active control treatment effect (exacerbation rate ratio) on the log_e_ scale.

Primary endpoint covariates: region, number of exacerbations in the prior year, baseline percentage predicted FEV_1_, pre-study biologic treatment for asthma and treatment group with log_e_(time in study in years) as an offset variable.

Secondary endpoint covariates: treatment group, region, number of exacerbations in the prior year, baseline % predicted FEV_1_, and pre-study biologic treatment for asthma.

Primary estimand (primary endpoint only): study discontinuation for reasons unrelated to the COVID-19 pandemic, change in maintenance therapy (excluding prohibited medication), and use of prohibited medication were handled using a treatment policy strategy. Study discontinuation for reasons related to the COVID-19 pandemic was handled using a hypothetical strategy.

Supplementary estimand (primary endpoint only): study discontinuation for any reason and use of prohibited medication were handled using a hypothetical strategy, and change of maintenance therapy was handled using a treatment policy strategy.

### Supplementary references

E1. FDA, Non-Inferiority Clinical Trials to Establish Effectiveness, 2016: <https://www.fda.gov/media/78504/download>
E2. Khatri, S; Moore, W, Gibson, PG, et al. Assessment of the long-term safety mepolizumab and durability of clinical response in patients with severe eosinophilic asthma. J. Allergy Clin Immunol. 2019;143(5):1742-51.
E3. Lugogo N, Domingo C, Chanez P, et al. Long-term efficacy and safety of mepolizumab in patients with severe eosinophilic asthma: a multi-center, open-label, Phase IIIb study. Clin Ther. 2016;38(9):2058-2070.e1.
E4. Ortega HG, Liu MC, Pavord ID, et al. Mepolizumab treatment in patients with severe eosinophilic asthma. N Engl J Med. 2014;371:1198-207.
E5. PASS 2020 Sample Size Software, NCSS.com. Tests for the Ratio of Two Negative Binomial Rates. Ch 438:1-17. Available at <https://www.ncss.com/wpcontent/themes/ncss/pdf/Procedures/PASS/Tests_for_the_Ratio_of_Two_Negative_Binomial_Rates.pdf>
E6. Pavord ID, Korn S, Howarth P, et al. Mepolizumab for severe eosinophilic asthma (DREAM): a multicentre, double-blind, placebo-controlled trial. Lancet. 2012;380:651-9.
