# Supplementary material for: Switching to twice-yearly depemokimab from mepolizumab/benralizumab in severe asthma: a multicenter, randomized, double-blind, phase 3A clinical trial (NIMBLE)
Source: Am J Respir Crit Care Med. 2026 Feb 11;212(5):921–35. doi: 10.1093/ajrccm/aamag031 (PMC13160935; doi:10.1093/ajrccm/aamag031)
Supplement: Supplementary file 1 [file aamag031_supplementary_data.zip › Supp Figure 1_HR.pdf]

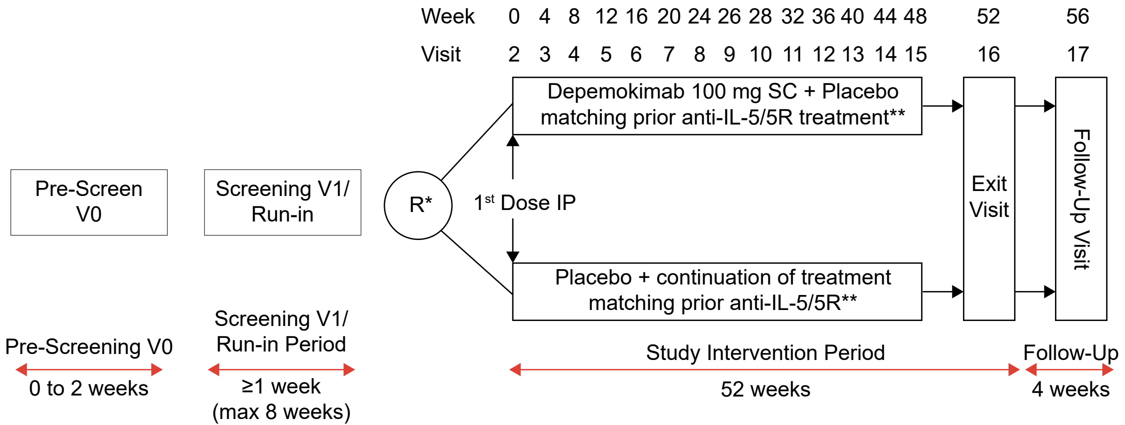

**R=Randomization*

***Participants randomized to depemokimab will receive placebo treatment matching their prior anti-IL-5/5R medication while subjects randomized to receive placebo matching depemokimab will receive active treatment matching their prior anti-IL-5/5R (either mepolizumab 100 mg every 4 weeks or benralizumab 30 mg every 8 weeks). All injections will be administered by a prefilled safety syringe (PFS)*
