# Supplementary material for: Switching to twice-yearly depemokimab from mepolizumab/benralizumab in severe asthma: a multicenter, randomized, double-blind, phase 3A clinical trial (NIMBLE)
Source: Am J Respir Crit Care Med. 2026 Feb 11;212(5):921–35. doi: 10.1093/ajrccm/aamag031 (PMC13160935; doi:10.1093/ajrccm/aamag031)
Supplement: Supplementary file 1 [file aamag031_supplementary_data.zip › Supp Figure 2_HR_EOS.pdf]

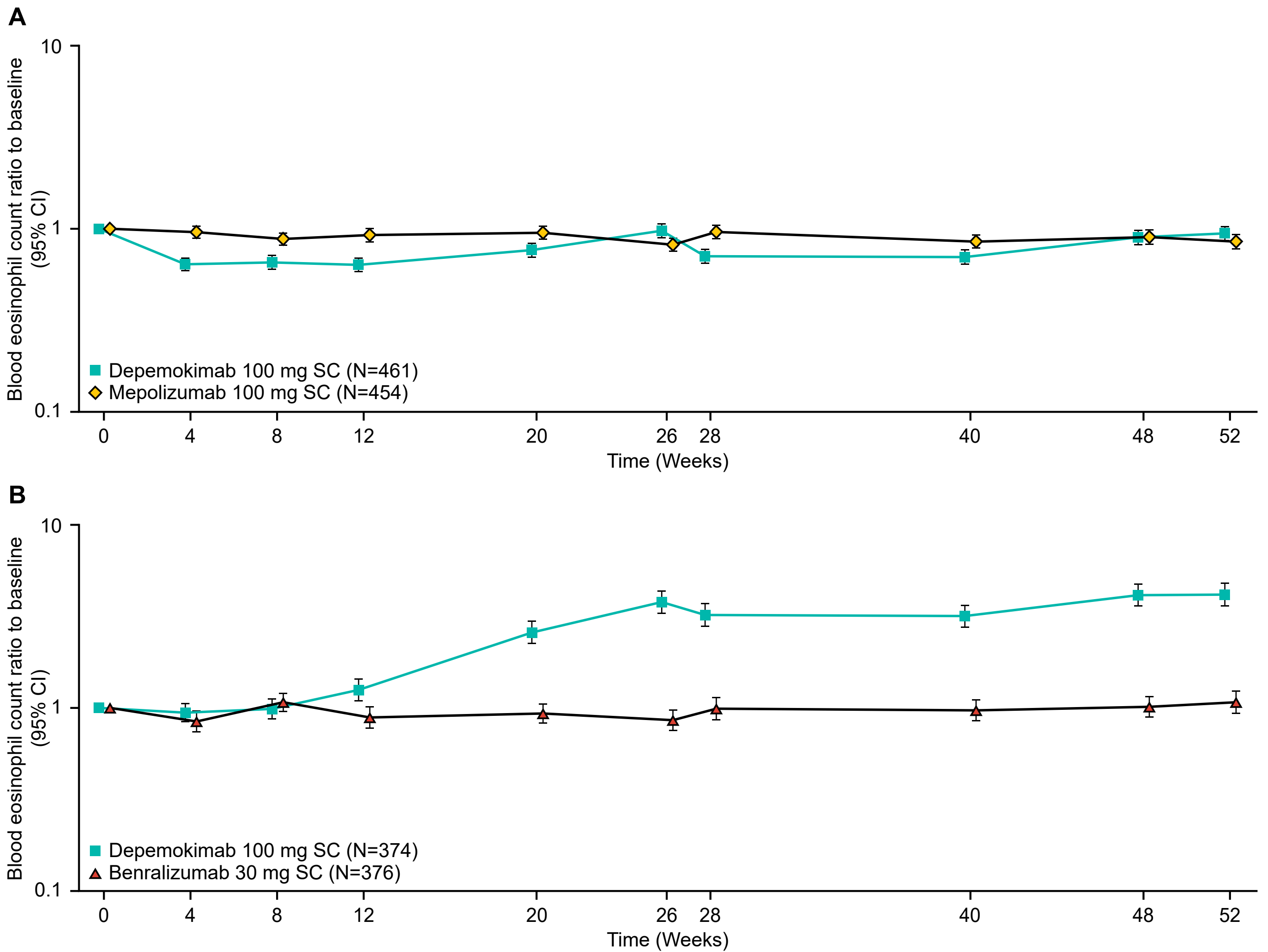
A
Blood eosinophil count ratio to baseline (95% CI)
10
1
0.1
Depemokimab 100 mg SC (N=461)
Mepolizumab 100 mg SC (N=454)
0
4
8
12
20
26
28
40
48
52
Time (Weeks)
B
Blood eosinophil count ratio to baseline (95% CI)
10
1
0.1
Depemokimab 100 mg SC (N=374)
Benralizumab 30 mg SC (N=376)
0
4
8
12
20
26
28
40
48
52
Time (Weeks)
